# New Evidences About Multidimensionality of the Patient-Practitioner Orientation Scale (PPOS) Construct in Undergraduate Medical Students

**DOI:** 10.1007/s40670-024-02119-4

**Published:** 2024-07-20

**Authors:** Marco Miniotti, Francesco Cuniberti, Alberto Olivero, Paolo Leombruni

**Affiliations:** ‘Rita Levi Montalcini’ Department of Neuroscience, University of Turin, Turin, Italy

**Keywords:** Patient-Practitioner Orientation Scale, Patient-centeredness, Doctor-patient relationship, Medical student, Factor analysis

## Abstract

**Background:**

Patient-centered care is becoming a paradigm in medicine. The Patient-Practitioner Orientation Scale (PPOS) is the only tool that measures the patient-centered attitude of healthcare students and professionals. Despite its spread, PPOS has had a poor process of scale modelling and validation and previous studies raised concerns about its psychometric robustness.

**Objective:**

This study aims to investigate the PPOS psychometric properties, factor structure, and construct validity on a large sample of undergraduate medical students.

**Methods:**

Participants of this cross-sectional single-center study are 1543 first-year medical students. PPOS item validity (internal consistency, test–retest reliability), factor structure (explorative principal axis factoring), and construct validity (convergent-discriminant validity, between-groups invariance) have been investigated.

**Results:**

A three-factor not clearly defined solution explaining 34.4% of the variance and containing 14 items out of 18 was retained. Internal consistency was questionable for factor 1 (*a* = 0.657), poor for factor 2 (*a* = 0.566), and unacceptable for factor 3 (*a* = 0.399). Item-total correlations for factor 1 and factor 2 were > 0.3, except for item 6 (ITC = 0.218) and item 12 (ITC = 0.283). Item total-correlations for factor 3 were all < 0.3. Test–retest reliability was acceptable for factor 1 (ICC = 0.704) and factor 2 (ICC = 0.789) and questionable for factor 3 (ICC = 0.661). Construct validity and measurement invariance across groups were satisfactory.

**Conclusion:**

Findings in this study corroborate previous evidences about PPOS psychometric limitations and provide new evidence about the multidimensionality of patient-centeredness construct.

## Introduction

Patient-centered care is becoming the most valued medical care approach worldwide. It focuses on patients’ preferences, needs, and values in medical procedures [1]. It has been defined through a multidimensional concept, consisting of five dimensions: the biopsychosocial perspective, patient-as-person, sharing power and responsibility, therapeutic alliance, and doctor-as-person [2]. This theoretical framework translates into a clinical practice in which the doctor, through his own communication and relational skills, is able to catch the patient’s issues, concerns, needs, beliefs, and meanings related to health and disease, in addition to symptoms and medical anamnesis. In this such way, it comes to be built a common ground of knowledge that allows for a concordance on disease etiology and treatment chances, and a sharing clinical decision-making, prevention, and health promotion [3]. Patient-centered care is proven to contribute to positive medical outcomes by promoting treatment adherence [4, 5], reducing the occurrence of complications linked to poor prognosis and adverse events [6], and enhancing self-management [7]. Moreover, it aims to improve patients’ emotional wellbeing and satisfaction for care [8, 9], and contributes to reduce healthcare costs through reductions in expenses related to diagnostic testing and specialist referrals [10].

Therefore, promoting and sustaining patient-centered care attitudes in future doctors has become a gold standard in medical education. However, a recent systematic review found that healthcare students have low attitudes toward patient-centered care [11]. As a result, evaluating the level of such attitudes among students is a pressing and foremost priority, followed by the development of educational interventions able to fill the gap. Among the instruments built to measure patient-centered care attitudes to date, the Patient-Practitioner Orientation Scale (PPOS) is one of the most specific [11]. The PPOS is an 18-item instrument in Likert format developed to capture healthcare students and professionals’ attitudes toward patient-centeredness [12].

The 18-item version of the PPOS is the result of multiple studies which lack some clarity as a whole. The initial PPOS version was developed using a pool of 35 potential items, drawn from an extensive search of the literature about patient-practitioner relationship, administered to a sample of undergraduate medical students. All the selected statements were written to refer to one of the two a priori postulated dimensions underlying the patient-centeredness construct, namely Sharing and Caring. The first reflects responders’ beliefs about sharing information and decision-making power with the patients. The seconds corresponds to the disposition to take care of them as “people,” respecting their expectations, feelings, and beliefs. The initial pool of items was progressively modelled eliminating one statement at a time based on item-total correlation value, calculated for each dimension separately, up to a 20-item scale [12]. A second study has been performed based on a different pool of 61 potential items administered to a sample of undergraduate psychology students. By using procedures similar to the above, 33 items were retained and the exploratory factor analysis (EFA) confirmed the scale construct as conceptually composed by the dimensions of Sharing and Caring [13]. Both the studies explored basic psychometric properties of the scale and claimed the tool was reliable based on good total internal consistency, with Cronbach’s alpha ranging from .80 to .90. In a third study performed on a mixed sample of physicians and patients, the PPOS was presented as an 18-item instrument, notwithstanding this further shortening was not documented in that nor in other studies available in the literature. In this latter study, the internal consistency observed was acceptable for the total scale and poor for the items pertaining to the Caring dimension. No other assessment of psychometric properties of the tool had been carried out [14].

Despite this questionable and poor process of scale modelling and validation, the 18-item version of the PPOS began to be used in the medical education literature. It has been translated into various languages and the validity and reliability of the different adaptations have been tested in multiple studies [15–21]. Some of these found the two-factor solution unstable and questioned the two-dimensional model [15, 18, 20]. Further studies suggested three-factor [21] and four-factor solutions [22].

Data suggesting the weakness of the PPOS two-dimensional model seem to confirm the multidimensional definition of the doctors’ patient-centered attitude [2], accordingly with the theoretical model proposed for patient-centered communication (consisting of four domains: patient’s perspective, psychosocial context, shared understanding, and sharing power and responsibility) [23].

In summary, patient-centeredness construct dimensionality, as measured by the PPOS, is far from clear and the distinction from other basic related constructs (e.g., empathy, attitude toward care) has not already been ascertained. This study aims to begin to fill this gap by investigating the PPOS psychometric properties, factor structure, and construct validity on a large sample of undergraduate medical students. In particular, this study aims to answer the following research questions: (Q1) Is the PPOS a psychometrically valid tool for assessing the patient-centeredness among first-year medical students? (Q2) Is the PPOS factor structure two-dimensional? (Q3) Is the construct measured by the PPOS significantly different between groups distinguished by gender, preferential interest, and setting toward medical practice?

## Methods

### Study Design, Procedures, and Participants

This cross-sectional study was conducted at the beginning of a lectures cycle on doctor-patient relationship and healthcare environment features. It targeted first-year students at the University of Turin Medical School during the initial half of the academic year between October and November. All the questionnaires used in the study (i.e., the Patient-Practitioner Orientation Scale, the Jefferson Scale of Empathy, the Frommelt Attitude Toward the Care Of the Dying Scale, and the Perceived Stress Scale) were administered two times during the first and the third lecture, with an interval ranged between 15 and 17 days. Students accessed online platform via tablets or computers in the classroom to complete the questionnaires. They were informed about purposes and methods of the study; participants voluntarily agreed to participate without any reward, and informed consent was obtained before they started filling out the questionnaire. Prior to questionnaires’ completion, students accessed the online platform through their university accounts. To maintain anonymity, they were required to enter a personal identification code. This procedure was implemented to ensure anonymity and facilitate questionnaire cross-matching. The study met the principles of the Declaration of Helsinki and obtained the ethical approval by the University of Turin Ethical Review Committee (No. 190231, 5 July 2014). Data gathering was repeated for three consecutive academic years with the same procedures in order to enlarge the sample size.

### Measures

At the beginning of the questionnaire, participants were asked to provide information about demographics and their interests and preferences toward medical profession (through the dichotomies “technical and interventional medical procedures” vs “humanistic and relational aspects of medical practice” and “caring for outpatients” vs “working in operating room or lab”).

### Patient-Practitioner Orientation Scale (PPOS)

The PPOS is a self-report questionnaire consisting of 18 items scored on a 6-point Likert-type scale (from 1 = “strongly agree” to 6 = “strongly disagree”) assessing attitudes toward patient-centeredness of healthcare students, physicians, and patients through two dimensions (9 items each): Sharing and Caring [12, 14]. Items are written in physician-oriented style, except items 6, 13, and 17. Scores of the scale and subscales are obtained by summing the items and dividing them by their corresponding number, so as the scores range between 1 and 6. The higher the score, the higher the attitude toward patient-centeredness. The 18-item Italian version of the PPOS developed by Ardenghi and colleagues was used in this study [20].

### Jefferson Scale of Empathy (JSE)

The JSE is a self-report questionnaire consisting of 20 items on a 7-point Likert-type scale (from 1 = “strongly disagree” to 7 = “strongly agree”) assessing empathic attitude in clinical practice of healthcare students and professionals [24]. The Italian translation provided by Jefferson Medical College, previously validated by the authors [25], was used in this study. JSE scores range from 0 to 140; the higher the score, the greater the empathic attitude.

### Frommelt Attitude Toward the Care Of the Dying (FATCOD) Scale

The FATCOD scale is a self-report questionnaire consisting of 30 items on a 5-point Likert-type scale (from 1 = “totally disagree” to 7 = “totally agree”) assessing the attitude toward the care of dying patients [26]. The Italian shortened version validated by the authors (FATCOD-9IT) was used in this study [27, 28]. FACTOD-9IT scores range from 9 to 45; the higher the score, the greater the aversion toward the care of the dying.

### Perceived Stress Scale (PSS)

The Perceived Stress Scale (PSS) is a self-report questionnaire consisting of 10 items on a 5-point Likert-type scale (from 0 = “never” to 4 = “very often”) measuring perceived stress [29]. The Italian version of the tool was used in this study [30]. PSS scores range from 0 to 40; the higher the score, the greater the level of the perceived stress.

### Visual Analog Scale (VAS) of Psychological Wellbeing

A VAS was used to assess psychological wellbeing. It consists of a horizontal line of 100 mm in length anchored by descriptors to each end (“negative wellbeing” at left, “positive wellbeing” at right). Responders have to indicate with a mark on the line the point that best represents their psychological state. Scores are determined by measuring in millimeters from the left end of the line to the marked point [31].

### Statistical Analyses

Data were analyzed with SPSS software v. 25.0 for Mac (IBM). Floor and ceiling effect for each PPOS item was investigated through the inspection of frequency tables, with a percentage ≥ 15% of respondents having scored the lowest or the highest point on the Likert-type scale considered indicative. PPOS factor structure was investigated through exploratory factor analysis (EFA) using the principal axis factoring (PAF) estimation method with orthogonal rotation and Kaiser-Meier-Olkin (KMO) measure of sampling adequacy > 0.70 (middling value) considered acceptable. Factors were extracted using the Kaiser-Guttman criterion (eigenvalues ≥ 1) and through the inspection of Cattell’s scree plot and the percentage of variance explained by the eigenvalues in the first round. Two fixed factors were extracted in the second round to simplify the factor structure (i.e., each variable should have one factor that clearly loads on it and each factor should clearly load on at least three variables), and improve its interpretability and understanding. Internal consistency of the scale was investigated by calculating Cronbach’s alphas and item-total correlations. Test–retest reliability was investigated through the inspection of intraclass correlation coefficients (ICC), with values ≥ 0.70 considered satisfactory. Robustness of construct validity was ascertained through the investigation of convergent and divergent validity and by testing a set of research hypotheses. Convergent-discriminant validity was investigated by calculating bivariate correlations (considering Pearson’s correlation coefficient) between each item of the PPOS and JSE, FATCOD-9IT, and PSS scores. It was expected that scales supposed to be conceptually related with PPOS items (i.e., JSE and, to a lesser degree, FATCOD-9IT, which is supposed to be conceptually related only with the Caring and not with the Sharing dimension) would be moderately positively correlated (i.e., Pearson *r* > 0.30). Conversely, it was expected that PSS, which is postulated to have little in common with PPOS items, would not be significantly correlated. Research hypotheses on between-group differences on PPOS score were developed and tested by specifying one-way multivariate analysis of variance (MANOVA) models with Scheffé post hoc test to address unequal group sizes, Cohen’s standardized effect size partial *η*^2^, and *α* set at 0.01. It was postulated that students who were females and had declared themselves more interested in humanistic and relational aspects of medical practice and prone to outpatient practice would score higher on the PPOS than those who demonstrated more interest toward technical and interventional medical procedures (e.g., lab test, surgery).

## Results

### Participant Characteristics and Scores

Participants in this study were 1543 first-year medical students at the beginning of their medical training. All the students approached in the classroom agreed to participate in the study (response rate = 100%), accounting for the 93.5% of the total cohorts resulting from the sum of the students enrolled in three academic years (*N* = 1650). Of the participants, 596 (38.6%) were males, 943 were females (61.1%), and 4 were non-binary (0.3%). The mean age was 19.7 ± 1.8. VAS for psychological wellbeing was 6.8 ± 2.0 (cm). Most of the participants stated they were more interested in technical and interventional medical procedures rather than in humanistic and relational aspects of medical practice (*n* = 776, % = 50.3 vs *n* = 601, % = 39.0) and they felt more prone to visit and care for outpatients rather than work in operating room or lab (*n* = 799, % = 51.8 vs *n* = 578, % = 37.5). A relevant number of participants (*n* = 166, % = 10.8) expressed no choice with respect to interest or preference for their future medical practice.

Participants scored 3.9 ± 0.5 on the PPOS, 106.5 ± 12.3 on the JSE, and 18.8 ± 6.3 on the PSS. Response rate for PPOS was 89.2%.

### Floor and Ceiling Effect

In this study, floor and ceiling effects correspond to having answered to a PPOS item “1 = strongly agree” and “6 = strongly disagree,” respectively. Floor effect ranged from 21.3% (item 5) to 40.1% (item 18). Ceiling effect ranged from 17.9% (item 8) to 74.9% (item 6). Full details are given in Table 1.

**Table 1:** Floor/ceiling effect and factor structure of the Italian version of the Patient-Practitioner Orientation Scale (PPOS)

PPOS item	% of scores		Factor loading				
	Low	High	F1	F2	F3	*h*^2^	ITC
7. If doctors are truly good at diagnosis and treatment, the way they relate to patients is not that important	0.9	41.6	**0.577**	0.060	0.061	0.340	0.428
16. It is not that important to know a patient’s culture and background in order to treat the person’s illness	0.3	52.4	**0.476**	0.065	0.033	0.232	0.356
2. Although healthcare is less personal these days, this is a small price to pay for medical advances	1.3	9.3	**0.446**	0.045	0.147	0.223	0.376
3. The most important part of the standard medical visit is the physical exam	6.1	1.8	**0.425**	0.141	0.057	0.203	0.405
11. If a doctors’ primary tools are being open and warm, the doctor will not have a lot of success	1.5	21.5	**0.421**	0.107	0.058	0.201	0.338
15. The patients must always be aware that the doctor is in charge	4.7	10.5	**0.413**	**0.376**	0.175	0.342	0.379
1. The doctor is the one who should decide what gets talked about during a visit	2.0	6.2	**0.326**	0.117	0.092	0.128	0.307
6. When doctors ask a lot of questions about a patient’s background, they are prying too much into personal matters	0.1	74.9	**0.324**	− 0.096	0.112	0.127	0.218
9. Patients should be treated as if they were partners with the doctor, equal in power and status	3.1	23.8				0.118	
13. A treatment plan cannot succeed if it is in conflict with a patient’s lifestyle or values	4.4	13.6				0.031	
18. When patients look up medical information on their own, this usually confuses more than it helps	40.1	0.9	− 0.051	**0.650**	0.099	0.435	0.416
5. Patients should rely on their doctors’ knowledge and not try to find out about their conditions on their own	21.3	4.0	0.084	**0.636**	0.082	0.418	0.458
12. When patients disagree with their doctors, this is the sign that the doctor not have the patient’s respect and trust	3.0	13.3	0.260	**0.358**	0.145	0.217	0.283^*^
10. Patients generally want reassurance rather than information about their health	7.6	4.9	0.042	0.141	**0.565**	0.341	0.277
17. Humor is a major ingredient in the doctor’s treatment of the patient	11.8	3.6	0.134	0.035	− **0.394**	0.174	0.180^#^
4. It is often best for patients if they do not have a full explanation of their medical condition	1.5	30.8	0.093	0.083	**0.358**	0.144	0.254
8. Many patients continue asking questions even though they are not learning anything new	2.8	17.9				0.156	
14. Most patients want to get in and out of the doctor’s office as quickly as possible	13.1	3.9				0.119	
% variance explained			9.9	7.0	5.0		
Cronbach’s			0.657	0.566	0.399		
ICC			0.704	0.789	0.661		

*Low/high*, % of lowest (1) and highest (6) possible scores; *F1/F2*, factor 1 and factor 2 extracted; *h*^*2*^, communalities; *ITC*, item-total correlation; *ICC*, intraclass correlation coefficient (test–retest reliability); *Cronbach’s *α* = 0.608 if deleted; ^#^Cronbach’s *α*=0.411 if deleted; Factor loadings >0.3 are reported in bold

### PPOS Factor Structure

Assumptions of sampling adequacy (KMO measure was 0.748, i.e., middling) and data factorizability (Bartlett’s test was χ^2^ = 2805.51, *p* < 0.001) were met. In the first round, with the Kaiser-Guttman criterion applied, EFA identified six factors with eigenvalues > 1, explaining 52.2% of the variance. The inspection of the scree plot showed two points of inflection at factors 3 and 4, suggesting to retain two or three factors. The two-factor solution explained 26.6% of the variance, with five items (9, 13, 4, 8, 17) non-retainable due to low loading (< 0.30). The three-factor solution explained 34.4% of the variance and showed slightly higher communalities and higher loadings than the former, with four non-retainable items (9, 13, 8, 14). Considering these little improvements, the solution found with Kaiser’s criterion and the large sample size, three factors were retained in the final second round of EFA. Table 1 shows factor loadings and communalities of the three-factor solution.

### Reliability Analysis

Internal consistency was questionable for factor 1 (*a* = 0.657), poor for factor 2 (*a* = 0.566), and unacceptable for factor 3 (*a* = 0.399). Item-total correlations for factor 1 and factor 2 were > 0.3, except for item 6 (ITC = 0.218) and item 12 (ITC = 0.283). Item total-correlations for factor 3 were all < 0.3. Test–retest reliability was acceptable for factor 1 (ICC = 0.704) and factor 2 (ICC = 0.789) and questionable for factor 3 (ICC = 0.661). Details of reliability analysis are provided in Table 1.

### Construct Validity

PPOS items were not strongly correlated with JSE, FATCOD-9IT, and PSS total scores. As expected for convergent-discriminant analysis, significant medium-to-weak correlations were found between PPOS items and JSE total score (except for items 4, 10, 14, 18), significant small correlations were found between few PPOS items (i.e., 6, 12, 14, 15, 15, 17) and FATCOD-9IT total score, whereas non-significant correlations were observed between PPOS items and PSS total score. Details of correlation analysis are provided in Table 2.

**Table 2:** Bivariate correlations of the Patient-Practitioner Orientation Scale (PPOS) items and sums of scores of the Jefferson Scale of Empathy (JSE), the 9-item version of the Frommelt Attitude Toward the Care Of the Dying (FATCOD-9IT) Scale, and the Perceived Stress Scale (PSS)

PPOS item	JSE	FATCOD-9IT	PSS
1	0.188**	0.019	− 0.011
2	0.298**	0.032	0.028
3	0.245**	0.012	0.046
4	0.047	0.062	0.025
5	0.097**	0.030	0.027
6	0.191**	0.110**	− 0.046
7	0.434**	0.048	− 0.084
8	0.143**	0.033	− 0.013
9	− 0.289**	0.015	0.078
10	0.023	0.071	− 0.019
11	0.324**	− 0.055	0.070
12	0.122**	0.095*	0.009
13	− 0.143**	− 0.044	− 0.040
14	0.032	0.120**	− 0.020
15	0.194**	0.077*	0.068
16	0.373**	0.090*	0.097
17	− 0.329**	0.081*	0.101
18	0.049	0.044	0.011

Pearson *r* < 0.30 indicates weak correlation; *p < 0.05; **p < 0.01

Research hypotheses developed about between-group differences on PPOS were confirmed. MANOVA showed significant effect of gender (*p* < 0.001), preferential interest (*p* < 0.001), and setting (*p* < 0.001) in medical practice on PPOS score. Female students and those who had declared themselves more interested in humanistic and relational aspects of medical practice and prone to outpatient practice scored higher on the PPOS than males and those who demonstrated more interest toward technical and interventional medical procedures (see Table 3). MANOVAs showed significant effect also of age (*p* = 0.007) and psychological wellbeing (*p* = 0.044) on PPOS score, with follow-up univariate analyses mostly non-significant (except for psychological wellbeing on factor 1). Details of MANOVA are provided in Table 3.

**Table 3:** Between-group differences on Patient-Practitioner Orientation Scale (PPOS) score

Variable	*Λ*	Part *η*^2^	F1	*p*	F2	*p*	F3	*p*
Gender	0.936**	0.033		< 0.001		0.001		0.060
Male			4.2±0.6		2.8±0.9		3.6±0.8	
Female			4.5±5.6		2.9±0.9		3.7±0.7	
Age group	0.987*	0.006		0.058		0.497		0.025
18–19			4.4±0.6		2.8±0.9		3.7±0.7	
20–21			4.4±0.6		2.9±0.9		3.7±0.7	
> 22			4.5±0.6		3.0±0.9		3.5±0.8	
Interest in medical practice	0.915**	0.085						
Doctor-patient relationship			4.6±0.6	< 0.001	2.9±0.9	0.139	3.7±0.7	0.023
Technical aspects and interventions			4.2±0.5		2.9±0.9		3.6±0.7	
Setting in medical practice	0.970**	0.030		< 0.001		0.186		0.516
Examination room			4.5±0.6		2.9±0.9		3.7±0.7	
Operating room/lab			4.3±0.6		2.9±0.9		3.6±0.7	
Psychological wellbeing (quartiles)	0.991*	0.005		0.010		0.891	q	0.409
Lower			4.5±0.6		2.9±0.9		3.7±0.7	
Middle			4.4±0.6		2.9±1.0		3.6±0.7	
Upper			4.3±0.6		2.9±0.9		3.6±0.7	

*Λ*, Wilks’ lambda; *p < 0.05; **p < 0.01; *Part η*^*2*^, partial eta squared; *F1/F2*, factor 1 and factor 2 extracted

## Discussion

Although the PPOS has been widely used in healthcare and medical education research in the last two decades, its psychometric properties have not yet been fully ascertained and its factor structure is still discussed. Since it is the only tool available to date to assess patient-centeredness attitudes of healthcare students and professionals, further research was required, also to shed more light on factors affecting doctors’ patient-centered behavior. In detail, the aim of this study was to investigate the PPOS reliability, factor structure, and construct validity on a large sample of undergraduate medical students. Taken together, results of this study showed that the PPOS has psychometric limitations when used to measure patient-centeredness among first-year medical students (Q1), the assumption of two-dimensionality for the scale construct is not respected in this population (Q2), and the scale adequately differentiates the patient-centeredness based on gender, preferential interest, and setting toward medical practice (Q3). These findings suggest that the PPOS 18-item version might contain items with poor validity and the construct it intends to measure is quite weak and unstable, as it changes when assessed in different populations. These results corroborate previous observations [18, 21, 22] and strengthen the need for scale revision [16, 20, 32].

### PPOS Construct Dimensionality: A Comparison with the Literature

Findings from reliability and item analysis suggest that PPOS includes a cluster of items with poor validity, having little variance in common, that are not very homogeneous each other, consistently with previous studies [18, 21, 33]. Findings about PPOS factor structure suggest multiple unclear not well separated solutions, as PAF pointed to possible six-, three-, and two-factor structures. The retained three-factor solution, which turned out to be the best in terms of percentage of variance explained and item numerosity observed in this study, nonetheless evidenced poor definition and functioning of many items, given low communality values and low factor loadings (items 9, 13, 8, 14 and were excluded from the final solution due to loadings < 0.30 and item 15 loaded on two factors), suggesting the weakness of scale construct. In the three-factor solution found in this study, F1 consisted of eight items (1, 2, 3, 6, 11, 15, 16) in partial overlap with the Caring dimension of the original PPOS, with the exception of items 1 and 15; F2 consisted of three items (8, 12, 15) all belonging to the Sharing dimension in the original PPOS; and F3 consisted of three items (4, 10, 17), of which the former two belonged to the Sharing dimension whereas item 17 belonged to Caring dimension in the original PPOS [14]. According to the PAF performed in this study, F1 seems to refer to the doctor’s relational style and disposition to be welcoming to the patient; F2 seems to refer to the doctor’s disposition to share power with the patient; and F3 seems to refer to the doctor’s disposition to be protective of the patient. F1 is the better-defined factor, more numerous and reliable, that matches the Caring dimension of the original PPOS, and is generally preserved in most studies [15, 17–21]. F2 and F3 were found to be less defined, numerous, and reliable. Items in these factors were observed to be mixed in previous studies [21, 22], suggesting that the Sharing dimension may be weaker and questionable. The items excluded from the final factor solution seem to be part of a critical and uncorrelated cluster with low ITCs and inter-item correlations; items 9 and 13, two of the three statements phrased in patient-oriented style, were excluded also in previous studies [15, 18, 21, 33], and items 8 and 14 were not retained in confirmatory factor analysis due to poor statistical indexes in PPOS shortening validation studies [20, 32]. Moreover, item 17, retained in this study even though its deletion would have increased F3 internal consistency, was deleted in multiple studies [18, 20, 21, 32, 33].

### PPOS Affordability Based on Medical Training

In addition to the documented psychometric weakness of the PPOS, one of the possible reasons behind the inconsistency of the construct the scale intends to measure could concern the instrument was designed and used to assess attitudes in population highly different in training, experience, and role, including healthcare students at different stages of their curricula (preclinical vs clinical), physicians, nurses, and patients. As correctly argued by Jiang and colleagues, understanding the concept of patient-centeredness and acknowledging the way of behaving in accordance with it is not the same as acting the attitude in clinical work [22]. In this study, first-year medical students asked to give an opinion on the statements of the PPOS, appeared to respond with confidence to the items of the Caring dimension, and seemed they were in trouble providing a personal evaluation on the items of the Sharing dimension. This should not be surprising, as while the Caring attitude reflects the purpose of the medical practice, in which the motivation to become a doctor of many young medical students is rooted, the Sharing attitude deals more with experience in clinical practice and the development of a personal clinical style. If so, the malfunctioning of F1 and F2 observed in this study might be due to its use with preclinical medical students.

### PPOS and Related Constructs

Findings from convergent-discriminant analysis showed the construct the PPOS measures correlates with empathy for patient care and attitude toward the care of the dying, while remaining distinct from them, and does not correlate with perceived stress, according with research hypotheses. Lastly, PPOS showed satisfactory measurement invariance across age and psychological wellbeing groups, according to research hypotheses.

### Limitations

This study has some limitations that must be mentioned. First, considering the lack of confirmatory analysis, findings about the PPOS three-dimensional model need to be confirmed in future studies. Second, the convenience sampling used in this study limits the generalizability of its findings. Third, the exclusive use of self-report measures does not allow to determine to what extent the data captured accurately reflects the participants’ real attitudes, beliefs, and future behaviors.

## Conclusion

Findings in this study support the conclusion that the 18-item version of the PPOS has psychometric limitations as regard reliability and item validity. Moreover, the robustness of the underlying construct is not proven. This means that it should be used with caution when assessing first-year medical students’ patient-centeredness. Further studies are needed to address these limitations, confirm the multidimensionality of scale construct, and ascertain that previous attempts of scale shortenings have not caused loss of content and original construct validity.
